# Acute Mesenteric Ischemia Secondary to Superior Mesenteric Vein Thrombosis

**DOI:** 10.7759/cureus.30819

**Published:** 2022-10-29

**Authors:** Shin T Zaw, Thinzar Zaw, Ahmad El-Far

**Affiliations:** 1 Medicine, Lake Erie College of Osteopathic Medicine, Bradenton, USA; 2 Medicine, University of Central Florida College of Medicine, Orlando, USA; 3 Hematology and Oncology, Winter Haven Hospital, Winter Haven, USA

**Keywords:** polycythemia vera, splenic vein thrombosis, portal vein thrombosis, jak2 mutation, superior mesenteric vein thrombosis

## Abstract

The thrombosis of the superior mesenteric vein (SMV) can result in ischemia of the intestine. A 71-year-old male presented with pain in the abdomen and a black tarry stool. The findings from computed tomography (CT) with the contrast of the abdomen suggest the thrombosis of the SMV. Heparin was administered, followed by thrombectomy and stenting of the SMV. The patient reported no complications and was shifted from heparin to apixaban and then discharged after a five-day hospital stay.

## Introduction

Acute mesenteric ischemia (AMI) is a sudden decrease in blood flow to a section of the intestine that causes ischemia, cellular damage, and intestinal necrosis [1]. It requires early treatment to prevent mortality. Superior mesenteric vein thrombosis (SMVT) accounts for 5-15% of total cases of AMI [1]. SMVT has a high mortality rate of 19-23%. A CT scan can identify 90% of the cases of SMVT [2]. This can be used for early detection of SMVT, and treatment with anticoagulants can be started. Here, we present a case of AMI due to SMVT.

## Case presentation

The patient is a 71-year-old man who presented to the emergency department after experiencing increasing acute and severe atraumatic pain in the abdomen. He reported experiencing diffuse, generalized abdominal pain and black tarry stools for several months. He has a past medical history of two previous strokes. He reported seven months ago that he had an upper and lower endoscopy performed with findings of Barrett's esophagus, hiatal hernia, and hemorrhoids. He is currently on aspirin 81 mg every day (QD). He has a family history of strokes, which contributed to his father's death. He stated that he was a previous pack, a daily smoker for 50 years, and currently uses recreational marijuana. He denied extensive alcohol intake.

**Figure 1:**
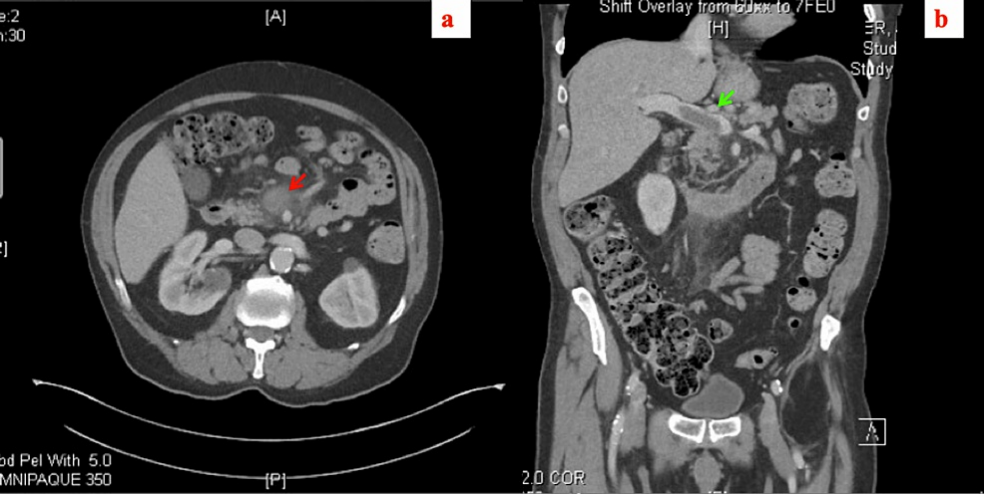
Computed tomography scan of the abdomen and pelvis with intravenous contrast: thrombus in the superior mesenteric vein with extensive perivascular infiltrates (red arrow) and partial obstruction of the portal vein (green arrow). (a) Axial view and (b) coronal view.

On examination, no bleeding was present. Laboratory investigations revealed a white blood cell count of 13.5 K/µL, hemoglobin concentration of 13.6 g/dL, RDW of 14.6, PT of 10.8 seconds, INR of 1.0, and PTT of 24.6 seconds. Urinalysis was unremarkable, and COVID-19 RT-PCR was negative. A CT of the abdomen and pelvis with contrast was obtained and revealed SMVT with extensive perivascular infiltration with partial occlusion of the main portal vein (Figure 1). The patient was started on an IV heparin drip for the management of SMVT.

**Figure 2:**
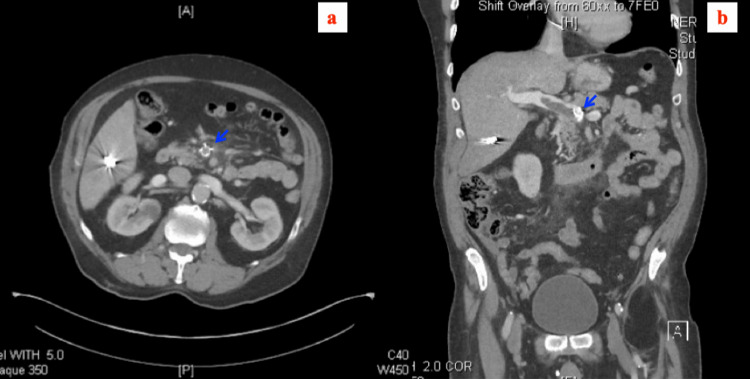
Computed tomography scan of the abdomen and pelvis with intravenous contrast: after placement of superior mesenteric vein stent (blue arrow). (a) Axial view and (b) coronal view.

Following the CT, interventional radiology was consulted. A portal venogram with thrombectomy, venoplasty, and stent placement of the SMV was performed without complications (Figure 2). Gastroenterology then performed an upper endoscopy to search for active bleeding, but the findings were unremarkable. Hematology instructed the patient to switch from a heparin drip to an apixaban drip for six months. Tests were ordered to screen for hypercoagulable states, and results indicated a negative flow cytometry test for paroxysmal nocturnal hemoglobinuria (PNH) and Janus kinase 2 (JAK-2) mutation. He was discharged in stable condition after a five-day hospital stay.

## Discussion

AMI is an abrupt cessation of blood flow to a portion of the small intestine that results in ischemia, cellular damage, and intestinal necrosis [1]. AMI accounts for about 0.09 to 0.2% of all acute admissions to the emergency department. AMI can be occlusive mesenteric ischemia or non-occlusive mesenteric ischemia [3]. The causes of mesenteric ischemia can be mesenteric artery embolism, mesenteric artery thrombosis, and mesenteric venous thrombosis (MVT), with an incidence of 50%, 15-25%, and 5-15%, respectively [1].

The SMV drains the intestine from the duodenum to the right two-thirds of the transverse colon and ends by joining the splenic vein to form the portal vein [4]. SMVT decreases blood drainage from this large portion of the gut and, therefore, can result in ischemic necrosis. The thrombosis of SMV can be either primary, i.e., without any predisposing cause, or secondary, i.e., due to known causes like thrombophilia, prothrombotic and primary hypercoagulability states (heparin-induced thrombocytopenia, essential thrombocythemia, post-splenectomy thrombocytosis, polycythemia vera, and neoplastic disorders) [2]. Singal et al. reported that the gain of function mutation in JAK2V617F is also associated with MVT [4].

Although the incidence of SMVT is low, the mortality rate is high, i.e., 19-23% [2]. This increases the importance of early diagnosis of thrombosis to prevent irreversible damage to the bowls. A CT scan is used for this purpose as it identifies 90% of these cases. The blood workup is usually not valuable for MVT, but some sources report an increase in D-lactate, lactate dehydrogenase, and L-lactate [2]. Abdominal Doppler ultrasonography is useful when there is blockage of large vessels. The incidence of MVT is high in males aged 40-60 years [4].

Patients with acute and sub-acute MVT may present with abdominal symptoms like severe mid-abdominal pain, nausea, gastrointestinal bleeding, constipation, and fever due to phlebitis [4]. Abdominal distention and ascites may also be present. In severe cases, rebound tenderness and rigidity may be reported due to peritoneal involvement [4]. Patients with chronic MVT are usually asymptomatic due to the development of collateral circulation and are diagnosed incidentally. The extent of ischemia and necrosis depends on where the blockage occurs. Most damage occurs when the terminal vessels are occluded. The development of ischemia and subsequent necrosis in MVT is gradual compared to that in mesenteric arterial thrombosis. The spasm of mesenteric arteries due to venous occlusion can occur, and this will result in no improvement in intestinal blood flow even after the venous blockage is removed [4].

Patients presenting with acute MVT are initially managed with analgesics, blood transfusion, and antibiotics [4]. Then anticoagulants are the preferred choice of management. Initially, unfractionated or low molecular weight heparin (LMWH) is administered, and the international normalized ratio (INR) is brought to 2-3. Then warfarin is administered. Due to the side effects of warfarin, direct thrombin inhibitors and direct factor Xa inhibitors are preferred. This regimen recanalizes the occluded veins [4]. A retrospective study by Hollingshead et al. involving 20 patients with MVT reported that the thrombolytic approach resulted in clot resolution in 75% of patients, 60% of patients reported complications, the most common of which was bleeding, and one patient died [5]. Zhang et al. reported in a retrospective study that the group of patients with MVT who received anticoagulant therapy had a shorter hospital stay, a reduced mortality rate, less need for surgery, and less chance of developing short bowel syndrome as compared to the group of patients that did not receive anticoagulant therapy [6]. Surgical management is given to patients presenting with peritoneal signs. Resection and anastomosis are done to preserve as much gut as possible [4]. Approximately 2-3 days after the first resection, another operation can be done to determine the need for another resection. Another study reported that 80% of patients with splanchnic vein thrombosis (SVT) were treated with anticoagulants, of which treatment with LMVH and vitamin K antagonists accounted for 31.9% and 25.4% of cases, respectively. Direct oral anticoagulants (DOAC) were used in only 1.7% of cases [7].

Our case reports a 71-year-old male with a history of smoking and a family history of stroke. He presented to the emergency department with acute abdominal signs and a black tarry stool. The diagnosis of acute mesenteric ischemia due to SMVT was made based on the findings of the CT abdomen. Heparin therapy was started, and thrombectomy, venoplasty, and stenting of SMV were performed. The patient was shifted from heparin to apixaban and was discharged after a five-day hospital stay, during which he reported no complications. The patient was found to be stable on apixaban therapy two months after being discharged from the hospital.

## Conclusions

AMI accounts for a minimal number of acute emergency cases but has a high mortality rate. Early diagnosis is now possible with advanced imaging technologies that can result in the early start of anticoagulant therapy, preventing irreversible damage to the intestines. The development of direct thrombin and direct factor Xa inhibitors provides a safe anticoagulant treatment of AMI secondary to SMVT.
